# Valorization of Spruce Bark to Environmentally Sustainable Packaging Materials

**DOI:** 10.1021/acssuschemeng.5c11166

**Published:** 2026-01-14

**Authors:** Houssine Khalili, Suthawan Muangmeesri, Lala Ramazanova, Léa Braud, Joseph S. M. Samec, Aji P. Mathew

**Affiliations:** † Department of Chemistry, 7675 Stockholm University, Stockholm SE-106 91, Sweden; ‡ Department of Sustainable Development, Environmental Science and Engineering, KTH Royal Institute of Technology, Teknikringen 10B, Stockholm 100 44, Sweden; § Stockholm University Centre for Circular and Sustainable Systems, Stockholm University, Stockholm SE-10691, Sweden

**Keywords:** spruce bark, lignin-containing microfibrillated cellulose, bark extractives, biobased films, and sustainable packaging, life cycle assessment

## Abstract

In this study, cellulose-based films were developed by using microfibrillated cellulose (MFC) and lignin-containing microfibrillated cellulose (Lig-MFC) derived from sequentially extracted spruce bark. The films were coated with the hydrophilic extractives from the fractionation, resulting in additional MFC-coated and Lig-MFC-coated cellulose films. A combination of morphological (AFM), surface (water contact angle (WCA), roughness), optical (UV–vis), and mechanical properties was analyzed to assess structure–property relationships. Coated films exhibited significantly enhanced hydrophobicity and UV-shielding, with WCA increasing from 47° to 76° for MFC and from 66° to 71° for Lig-MFC. Notably, MFC-coated films displayed superior mechanical performance, with a tensile strength of 119 MPa and elongation of 11%, surpassing most lignocellulosic-based films in the literature derived from bark. In addition, this tensile strength falls within or above the range of commonly used materials, such as kraft liner, PET, and LDPE, suggesting realistic opportunities for substitution in short-lived packaging applications. AFM analysis revealed a reduction in surface roughness after coating, correlating with an enhanced WCA. Compared with similar biobased films in the literature, the extractive-coated MFC films show superior performance in terms of strength, flexibility, and UV-shielding properties. This valorization route offers both economic and environmental sustainability advantages compared with incineration for energy recovery. A comparative life cycle assessment (LCA) study showed that valorization of the pulp and hydrophilic extractives from the bark biorefinery into different qualities of MFCs gave substantial climate change benefits stemming from the possibility of substituting packaging materials with high inherent environmental impact.

## Graphical abstract

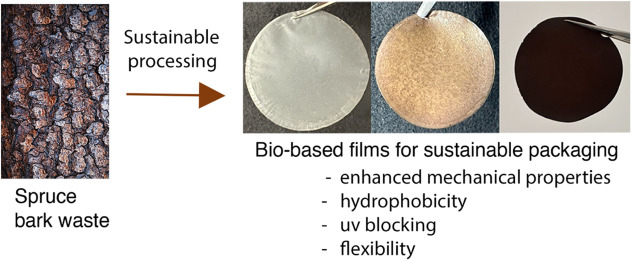

## Introduction

Spruce bark is a major byproduct from sawmills and pulp mills, generating approximately 7.7 million m^3^ annually only in Sweden.^1^ Bark is currently incinerated for heat and electricity, which is considered carbon neutral; however, it is not climate neutral. Spruce bark contains valuable lignocellulosic components, including hydrophilic extractives that are not present in wood. These components can be valorized into high-value-added products, offering both economic and environmental advantages.

Lignocellulosic biomass can be fractionated into cellulose, lignin, and extractives through a range of chemical processes.^2,3^ Ek and coworkers have previously demonstrated that nanocellulose can be produced from spruce bark; however, the mechanical properties were not evaluated.^4,5^ In our previous work, we showed that spruce bark can be sequentially extracted under mild conditions in a continuous flow system, yielding high-purity fractions and enabling environmentally beneficial valorization pathways.^6,7^ These biobased components were proposed as promising for sustainable material applications.^8^

The intrinsic properties of each component contribute to final material performance, but designing sustainable materials requires minimizing chemical modification, energy consumption, and processing cost. Retaining lignin in cellulose fibers by avoiding full bleaching reduces energy use and preserves lignin’s natural functionalities.^9^ Mechanical fibrillation of unbleached biomass produces lignin-containing microfibrillated cellulose (Lig-MFC), where residual lignin can facilitate fibrillation and increase yield.^10^ Several studies highlight lignin’s multifunctionality in Lig-MFC systems, including contributions to barrier performance, hydrophobicity, and UV shielding.^11–13^ Lignin-rich fibrils (>15 wt %) have also shown enhanced emulsification behavior, improved compatibility with hydrophobic polymers, and increased mechanical robustness in composite materials.^14,15^ Additionally, lignin’s aromatic chromophores impart strong UV-blocking capability, broadening its potential for functional coatings.^16–18^

In addition to the components found in spruce wood, bark contains significant amounts of hydrophilic and lipophilic extractives such as resin acids, simple sugars, and tannins.^19^ These extractives have been used in applications such as tanning agents, bioadhesives, antioxidants, antimicrobials, UV-protective formulations, fungicides, and pharmaceuticals.^20–22^ However, studies integrating bark extractives directly into cellulose-based materials remain limited. Prior work has largely focused on producing cellulose or lignocellulosic fibers from bark or incorporating extractives as external additives. For instance, bark-derived nanofibrils have been used in protective coatings,^23^ while other studies combined willow bark extract with modified birch nanocellulose using UV or enzymatic cross-linking.^24^ Bioactive foams incorporating cellulose and willow bark extracts have also been reported for antioxidant and UV-protective functions.^25^

Here, we present a bioinspired approach in which both the structural (MFC and Lig-MFC) and functional (hydrophilic extractive) fractions originate from the same spruce bark source. We demonstrate that extractives can act as a natural coating for mechanically defibrillated Lig-MFC and MFC films, enabling fully bark-derived packaging materials with improved mechanical strength, hydrophobicity, and UV shielding, all achieved under mild conditions without chemical modification or heat treatment. This simple, low-cost strategy links waste valorization with sustainable material design. Finally, a comparative life cycle assessment (LCA) shows that producing these two MFC qualities offers measurable climate benefits compared with the current practice of bark incineration.

## Materials and Methods

### Materials

The bark was provided by Södra Skogsägarna. All chemical reagents were purchased from Fisher Scientific, CCS Healthcare AB, Sweden, Sigma-Aldrich, Honeywell, and VWR Chemicals and were used as received. PVDF membrane with a pore size of 0.45 μm was purchased from Durapore, bovine serum albumin (BSA) lyophilized powder ≥96% with a Mw of 66 kDa (40 × 140) was purchased from VWR, and phosphate buffered saline (PBS) pH 7.2 was purchased from Sigma-Aldrich. Ethanol 95% was acquired from Solveco. All chemical handling should be thoroughly assessed with respect to safety before running experiments.

### Preparation of the Material

#### Compositional Analysis of Spruce Bark

Spruce bark (5 g) was heated overnight at 60 °C to measure the moisture content. Lipophilic components were extracted in a Soxhlet extractor using ethyl acetate (150 mL) at 100 °C for 6–8 h. The resulting liquid was filtered and concentrated to yield lipophilic extractives that were subjected to GC–MS analysis. The solid residue was oven-dried overnight at 60 °C and subjected to the second extraction in a Soxhlet extractor with 150 mL of water for 2 h. The aqueous solution was filtered and concentrated to yield noncellulosic sugars, which were subjected to the HPLC analysis to determine their composition. The remaining solid residue was oven-dried and further analyzed for its chemical composition by using the Klason method. For sugar analysis, the analysis was conducted using an Agilent 1200 Series HPLC system equipped with a Biorad Aminex HPX-87P column (300 × 7.8 mm) and coupled with a refractive index detector.

#### Sequential Extraction of Lipophilic and Hydrophilic

A total of 10 g of spruce bark was subjected to two extraction steps in an autoclave reactor. The process began with an extraction using 200 mL of ethyl acetate at 100 °C for 6–8 h, followed by a second extraction with 200 mL of water at 120 °C for 2 h. These sequential extractions were designed to remove lipophilic and hydrophilic compounds, respectively, and gave 70 mg of lipophilic extractives, 78 mg of hydrophilic extractives, and 9.85 g of extractives free bark.

#### Soda Pulping

5 g of pretreated bark described above was treated with 200 mL of 10 g/L NaOH in an autoclave. The reaction was conducted at 200 °C for 2 h. Upon completion, the reaction mixture underwent filtration, and the collected liquor was precipitated using H_2_SO_4_ to isolate the lignin. The resulting precipitate was filtered. Following this step, the precipitate was analyzed using GPC. The solid pulp was thoroughly washed with water and dried in an oven at 60 °C overnight to yield 3.6 g. Subsequently, the pulp was prepared for chemical composition analysis. Lignin was dried and isolated yielding 1.2 g.

#### Bleaching of Pulp

To improve the brightness, the pulp underwent chlorite bleaching. The bleaching mixture was prepared using 2 g of pulp in a total volume of 200 mL, consisting of equal portions (100 mL each) of two solutions: (i) 1.7% sodium chlorite (NaClO_2_) and (ii) 2.7% sodium hydroxide (NaOH) with 7.5% acetic acid (AcOH). The treatment was carried out at 80 °C for 2–3 h and repeated 2–3 times. Following bleaching, the pulp was filtered and thoroughly washed with water.

#### Preparation of Lig-MFC and MFC

Materials for Lig-MFC and MFC were prepared from unbleached soda pulp and bleached pulp, respectively. Initially, each pulp was pretreated using an Ultra-Turrax mixer for approximately 30 min. This was followed by mechanical fibrillation by using a microfluidizer. The soda pulp was processed at a concentration of ∼1 wt %, while the bleached pulp was processed at 0.5 wt %. The microfluidizer was operated with a channel size of 100 μm and a pressure of 1500 bar. Lig-MFC was obtained after 9 passes, whereas MFC required 20 passes.

#### Film Processing

The previously prepared materials were used for the film fabrication. First, the pristine Lig-MFC film was prepared by vacuum-filtering a Lig-MFC suspension onto a PVDF membrane (0.45 μm), followed by drying at room temperature under a weighted load. To prepare the Lig-MFC film coated with extractives, a fresh Lig-MFC suspension was first filtered, followed by filtration of the extractive suspension directly onto the film. The resulting layered film was then dried under the same conditions as for the pristine film. The extractive suspension was prepared by dissolving freeze-dried extractives in a 10:1 ethanol-to-water solution. Filtration proceeded rapidly, and the final film had a thickness of approximately 39–78 μm. A similar procedure was applied to prepare the MFC film (see Figure 1).

**Figure 1:**
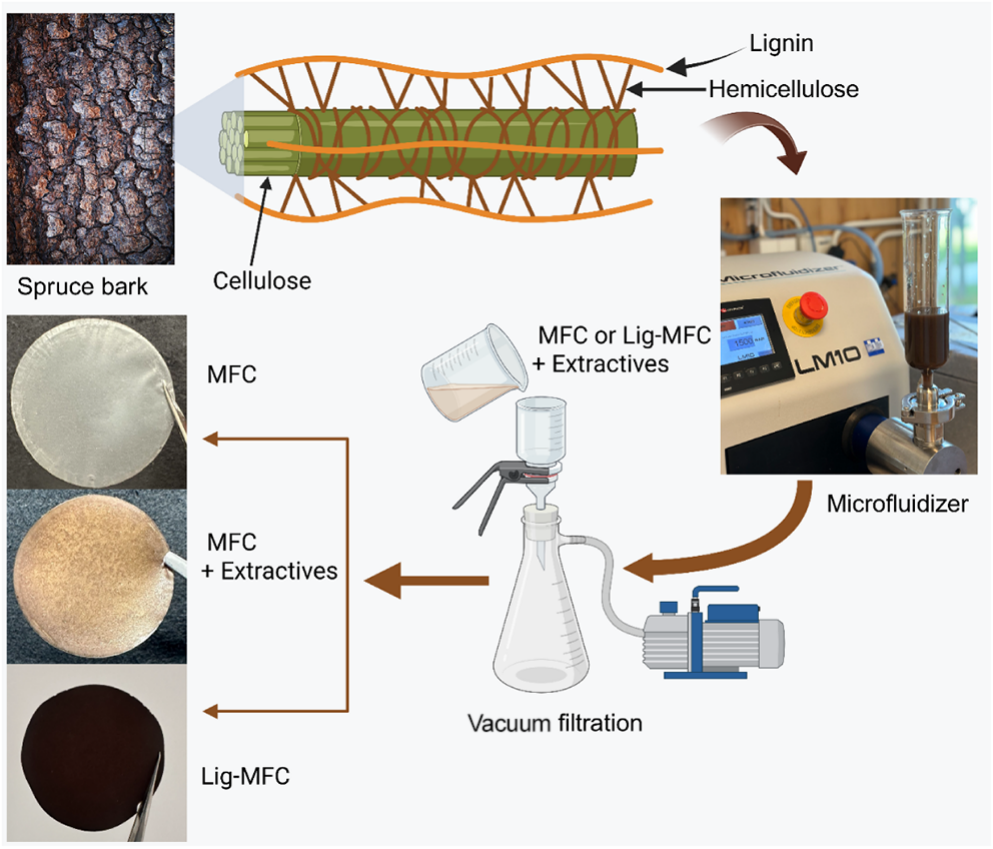
Schematic of the spruce bark valorization process and film preparation. Spruce bark is fractionated to obtain pulp for microfibrillated cellulose (MFC) and lignin-containing microfibrillated cellulose (Lig-MFC), which are mechanically fibrillated by using a microfluidizer. Films are formed by vacuum filtration and coated with bark extractives. Representative images of the MFC, Lig-MFC, and extractive-coated films are shown. This figure is created in BioRender. Khalili, H. (2025) https://BioRender.com/r1qv1zz.

### Characterization Techniques

#### X-ray Diffraction

The semicrystalline structure and the crystallinity of the prepared materials were examined using the D8 Discover powder diffractometer, operated at a voltage of 40 kV and a current of 40 mA, with monochromatic CuKα radiation (λ = 0.154 nm). The crystallinity index (CrI) was evaluated according to the Segal equation^26^

$$\mathrm{CrI}\;\left(\%\right)=\frac{I_{200}-I_{\mathrm{am}}}{I_{200}}$$

where *I*_200_ is the intensity of the 200-lattice plane at around 2θ = 22.56°, and *I*_am_ is the intensity from the amorphous phase at approximately 2θ = 18.9°.

#### Fourier Transform Infrared Spectroscopy

The chemical structure of all the prepared materials was characterized using Fourier transform infrared spectroscopy (Varian 670-IR spectrometer) equipped with an ATR accessory. Each spectrum was recorded in the range of 4000 to 600 cm^–1^, with a resolution of 4 cm^–1^ and an accumulation of 128 scans.

#### Atomic Force Microscopy

A Dimension Icon AFM instrument (equipped with a Nanoscope controller, Bruker) was used in the PeakForce Tapping mode with a TESPA-V2 probe (spring constant, *k* = 42 N/m). Prior to imaging, the suspensions were diluted to 0.001 wt %, sonicated, and spin-coated onto freshly cleaved and APTES-coated mica substrates, which were affixed to AFM metal discs using double-sided tape. The nanomechanical properties of the materials were evaluated using PeakForce Quantitative Nanomechanics (PFQNM) mode, employing the same probe and sample preparation protocol. The spring constant was calibrated using the thermal tune method, and the deflection sensitivity was determined using the Sader method (cantilever width and length were measured with an optical microscope). The nominal tip radius was used for all measurements. Data were processed using Nanoscope Analysis software to measure the height of at least 100 fibers for better statistics. Surface roughness of the films was also measured using the same instrument and probe, with the films mounted on lamella glass slides using double-sided tape (with representative corresponding PeakForce images in Figure S4).

#### Water Contact Angle

The contact angle of the films was measured using a Drop Shape Analyzer DSA25E (Krüss Scientific). Each film sample was affixed to a glass slide by using double-sided tape to ensure a flat surface. Measurements were performed using the sessile drop method with 2 μL of deionized water droplets, and the contact angles were automatically calculated using ADVANCE software (Krüss Scientific).

#### UV–Visible Spectroscopy

The optical transparency of the prepared films was measured by using UV–visible spectroscopy (Agilent Cary 5000 UV/vis/NIR spectrometer) operated in transmission mode. Rectangular film samples were placed directly in the spectrophotometer test cell, and air was used as the reference. The optical transmittance of the films was recorded in the wavelength range of 200–800 nm.

#### Tensile Test

Tensile properties of the films were evaluated according to ASTM D882-00 using a universal testing machine (Instron 5960 dual-column tabletop) equipped with a 1 kN load cell. Film samples were cut into rectangular strips with a fixed gauge length of 10 mm and a crosshead speed of 5 mm/min. All tests were performed in triplicate, and the reported results represent the average values.

#### Environmental Life Cycle Assessment

A comparative consequential LCA was carried out following ISO 14040:2006 and ISO 14044:2006 standards to evaluate the environmental consequences of valorizing bark via a novel biorefinery process versus its incineration. The incineration of bark for heat and power production represents the baseline scenario and serves as a benchmark in this study.

A functional unit (FU) of 1 kg of bark dry weight was used, thereby focusing the assessment on the conversion pathways rather than the specific biorefinery products. This work builds on a preliminary environmental sustainability assessment that indicated the potential benefits of bark biorefining compared with its conventional use as an energy feedstock.^7^ Here, the focus is on evaluating whether an additional processing step to convert part of the pulp and hydrophilic extractives into coated Lig-MFC and coated MFC affects the overall environmental performance of the biorefinery.

The system boundaries are defined as the cradle-to-biorefinery-gate, including all processes up to the production of the biorefinery outputs. No data are currently available on the incorporation of Lig-MFC and MFC into end products, and these downstream stages are excluded from the system. The biorefinery process yields six valuable fractions: lipophilic extractives, hydrophilic extractives, lignin, pulp, Lig-MFC, and some residual biomass. Following similar assumptions as in a previous study,^7^ and in the absence of detailed data, it was assumed that these fractions are further converted into tall oil, starch, phenol, sulfate pulp, material-grade Lig-MFC, and heat, respectively. Given the consequential modeling framework and input-related FU, multifunctionality was addressed by applying substitution to all coproducts, without designating a main product or function. Substitution factors for Lig-MFC and MFC are derived from their tensile strengths relative to displaced packaging materials: kraft liner (corrugated box linerboard, 20–40 MPa), polyethylene terephthalate (PET) film (50–75 MPa), and low-density polyethylene (LDPE) film (10–15 MPa). This ensures equivalent functional performance rather than mass equivalence, a physically justified approach for structural packaging applications, where mechanical properties govern material efficiency. Detailed calculations are provided in SI-2.

The coated Lig-MFC and coated MFC were assumed to be used in the production of sustainable packaging materials. Biobased packaging materials frequently demonstrate a shorter service life than their fossil-based equivalents.^27^ However, this study provides a preliminary environmental feasibility assessment of the biorefinery from cradle-to-gate, excluding the use phase and end-of-life stages of the packaging due to the novelty of the process. The baseline scenario assumes that all pulp and hydrophilic extractives from the biorefinery substitute equivalent market volumes of pulp and starch, in alignment with the selected functional unit. A dedicated follow-up LCA, contrasting Lig-MFC-based packaging with fossil-derived alternatives, will be required at later development stages when superior data are accessible.

Climate impacts of the incineration and biorefinery scenarios were quantified using the IPCC 2021 life cycle impact assessment (LCIA) method with a 100-year time horizon.^28^ In addition, the ReCiPe midpoint hierarchist (H) v1.1 method was used to assess 17 additional impact categories, with particular attention to eutrophication, particulate matter formation, human toxicity, and terrestrial acidification.^29^ These impact categories were selected to capture the key effects associated with bark incineration and with solvent- and energy-intensive biorefinery operations.

The foreground system was parameterized using experimental data for Lig-MFC and MFC production generated in this work, complemented by data from a previous study for the remaining biorefinery processes (see SI-2). The background system is modeled using the ecoinvent v3.9 consequential database,^30^ and all calculations are performed with the Brightway LCA framework.^31^ A full description of the life cycle inventory (LCI) is provided in SI-2, and the Python scripts used for the LCA modeling are available in an open-source GitHub repository.

## Results and Discussion

### Sequential Extraction of Spruce Bark

The compositional analysis of spruce bark disclosed the following composition: moisture, 6%; lipophilic extractives, 2%; noncellulosic sugars, 16%, (mainly glucomannan) and other components, 19%; cellulose, 20%; hemicellulose, 10%; lignin, 32%; ash, 1% (Figure 2).

**Figure 2:**
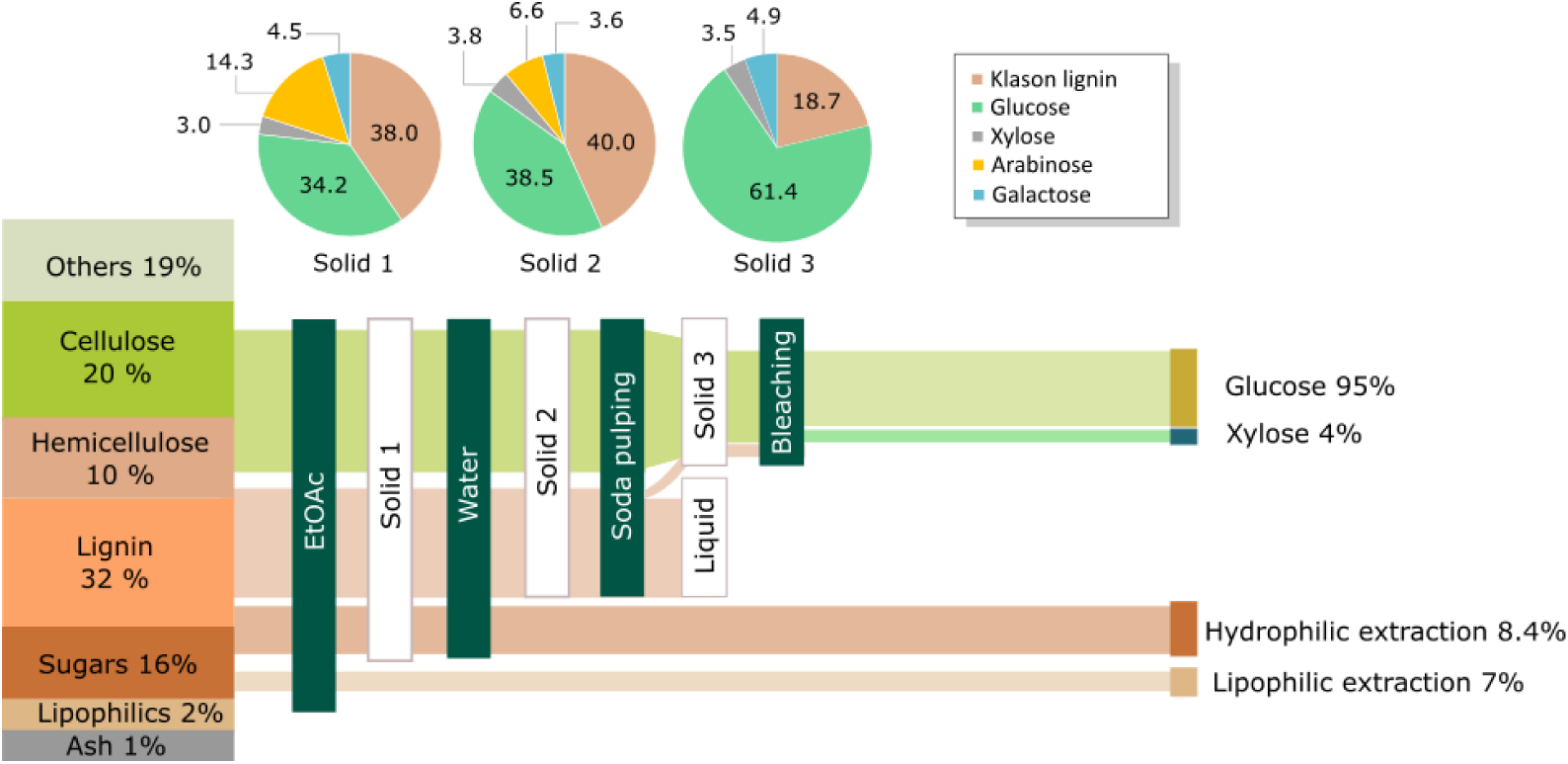
Sequential fractionation of spruce bark to obtain a hydrophilic fraction to be used as a coating reagent, unbleached pulp for Lig-MFC, and bleached pulp for MFC.

The sequential extraction was performed on a 10 g scale. Lipophilic extraction removed 7 wt % of the total mass, indicating that small amounts (5%) of other components were also extracted (Figure 2). This fraction was analyzed by GC–MS and revealed terpenes and fatty acids (Figure S3). Hydrophilic extraction gave 8.4 wt % of noncellulosic sugars comprising pectin-like substances (Figure S2) that will be used as a coating formulation to substitute starch, *vide infra*. The remaining residue comprised almost equal amounts of lignin (40%) and glucose (38.5%) and 14% of other sugars, mainly hemicelluloses. Soda pulping was performed on a 5 g scale to produce 3.6 g of unbleached pulp and 1.2 g of lignin. Compositional analysis of the pulp revealed 61.4% glucose and 18.7% lignin. This fraction was used in the next step to produce Lig-MFC. The unbleached pulp (2.0 g) was also bleached using sodium hypochlorite to generate the bleached pulp in 75% yield (1.5 g). To build up the inventory for the LCA, optimized mass and energy balances from a previous report were used,^6,7^ which represent an industrial process better. Yields from bleaching experiments were used; however, a simulated industrial bleaching process from ecoinvent was used.

### Structural Characteristics of Lignin-Containing Microfibrillated Cellulose and Extractives

FTIR was used to confirm the presence and absence of lignin in both Lig-MFC and MFC and also to identify the functional groups existing in the materials (Figure 3a). In the MFC material, a broad band at 3317 cm^–1^ was associated with the O–H stretch of the hydroxyl in lignocellulosic biomass, which is also present in the material Lig-MFC.^32^ Furthermore, the band located at 2900 cm^–1^ for MFC is assigned to the C–H stretching, which is slightly shifted to 2916 cm^–1^ for Lig-MFC. The band characteristic of lignin aromatic skeletal vibration is present at 1569 cm^–1^ in the Lig-MFC sample but disappears in the MFC, indicating successful removal of lignin after the bleaching step.^33^ The band at 1018 cm^–1^ in MFC is attributed to the C–O–C glycosidic bond, while the band at 1028 cm^–1^ in Lig-MFC corresponds to C–O of primary alcohol or guaiacyl C–H.^34^ As far as the extractives fraction obtained from Spruce bark are concerned, the FTIR spectrum shows characteristic bands at ∼3300  cm^–1^ (O–H stretching), ∼2900  cm^–1^ (C–H stretching), ∼1600–1510 cm^–1^ (aromatic C=C stretching), and a strong band at ∼1025  cm^–1^, corresponding to C–O stretching vibrations in alcohols in polysaccharides. These features confirm the presence of hydroxyl-rich sugars such as glucose, xylose, arabinose, and galactose, as also verified by HPLC. However, the poor solubility of the extract in water and its full dissolution only in a 10:1 ethanol:water mixture indicate that the fraction likely contains aromatic or phenolic compounds with limited polarity.^35^

**Figure 3:**
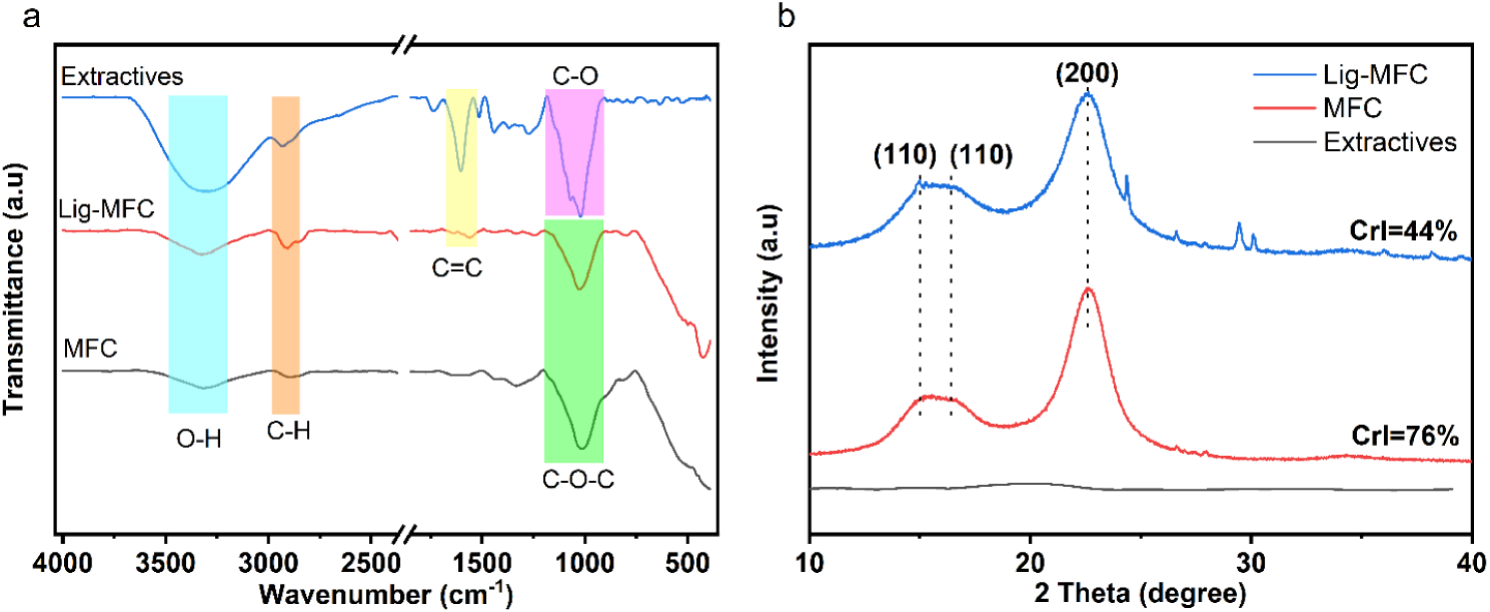
(a) FTIR spectra and (b) XRD patterns of MFC, Lig-MFC, and the bark extractives used for coating. The FTIR spectra highlight characteristic O–H, C–H, C=C, and C–O–C bands, while the XRD profiles show cellulose Iβ reflections and corresponding crystallinity indices for MFC and Lig-MFC.

The XRD patterns for MFC and Lig-MFC show the characteristic peaks of cellulose Iβ. The broad peak at 15.7° consists of a mixture of two peaks corresponding to the Miller indices (110) and (110), while the peak at 22.56° and the peak at 34.9°, which is not clear, correspond to (200) and (004), respectively.^36^ The Segal equation was used to calculate the relative amount of the crystalline fraction of cellulose. The results indicate an increase in the index from Lig-MFC to MFC, which is mainly due to the removal of lignin that contributes to the amorphous background. As far as the extractive is concerned, the pattern indicates that it is an amorphous material (see Figure 3b).

### Atomic Force Microscopy

AFM imaging along with fiber height histograms revealed some morphological differences between the MFC and Lig-MFC films (Figure 4). Both samples displayed quite similar height distributions. However, the Lig-MFC was obtained after 9 passes, while the MFC was obtained after 20 passes. Some previous work suggests that the presence of residual lignin contributes to improved fibrillation efficiency by interfering with fiber–fiber reaggregation during mechanical disintegration. Rojo et al. observed reduced fibril diameters (down to 16 nm) in lignin-containing cellulose nanofibers, attributed to the radical scavenging role of lignin during high-shear processing.^12^ The Lig-MFC showed the appearance of some particles, which are most likely lignin particles. These later appear to be attached to the fibers or free on the mica substrate (see Figure 4b and Figure S1). To further investigate the local mechanical behavior of the films, quantitative nanomechanical mapping was performed by using atomic force microscopy. The measured surface modulus of the MFC film was 1.04 ± 0.11 GPa, while the Lig-MFC film exhibited a slightly lower modulus of 0.82 ± 0.06 GPa. The higher modulus in the MFC is attributed to its higher crystallinity and more uniform fibrillar packing, while the presence of residual lignin in the Lig-MFC may contribute to a more heterogeneous and compliant surface structure. These results align with previous reports indicating that lignin-containing nanofibers often exhibit reduced stiffness due to the amorphous and flexible nature of lignin (see modulus maps in Figure S4).

**Figure 4:**
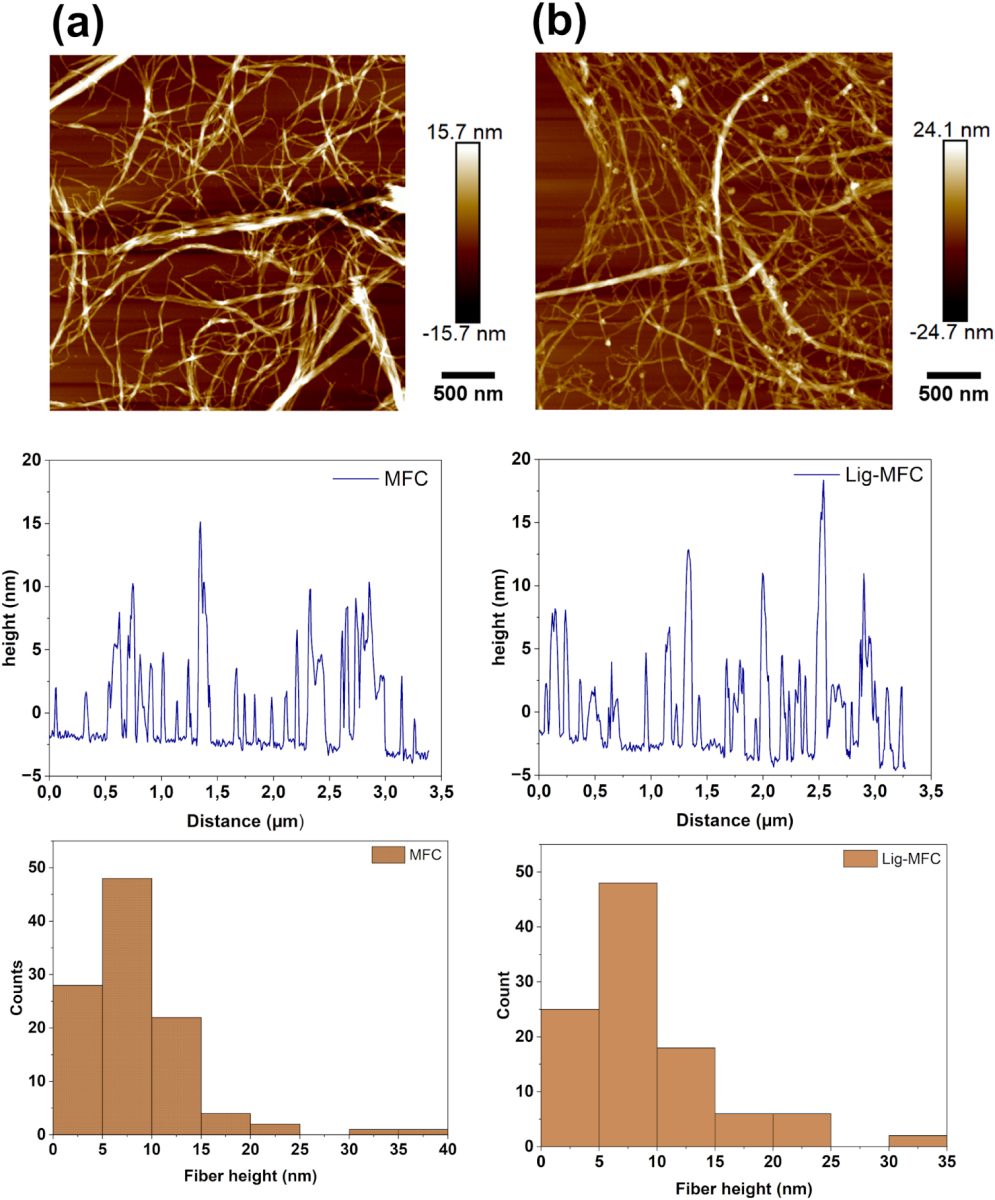
AFM height images of (a) MFC and (b) Lig-MFC, shown with corresponding height profiles and fiber-height distributions. The images illustrate the nanoscale fibrillar morphology and the broader height distribution observed for Lig-MFC due to residual lignin.

### Optical and Surface Characteristics of the Films

The optical transmittance property of lignocellulosic-based films is critical for applications in sustainable packaging and UV-shielding materials. In this study, four different films were evaluated for UV–vis transmittance: MFC, Lig-MFC, and their extractive-coated counterparts.

The results in Figure 5a show that the MFC displayed the highest transmittance in the UV–visible range, consistent with the absence of light-absorbing compounds. In contrast, Lig-MFC showed strongly reduced transmittance in the visible region and almost zero transmittance in the UV region, which can be attributed to the lignin’s intrinsic chromophore, in particular phenolic, carbonyl, and aromatic groups that strongly absorb light between 200 and 400 nm and partially into the visible region depending on the lignin content.^18^ This finding aligns with Dou et al. who reported excellent UV shielding for willow bark-derived LCNF films and lower visible light transmittance compared to MFC films.^37^ Interestingly, Almeida et al. observed cases where residual lignin improved transparency due to more uniform fibril morphology after cationic or enzymatic pretreatments, reducing light scattering.^38^

**Figure 5:**
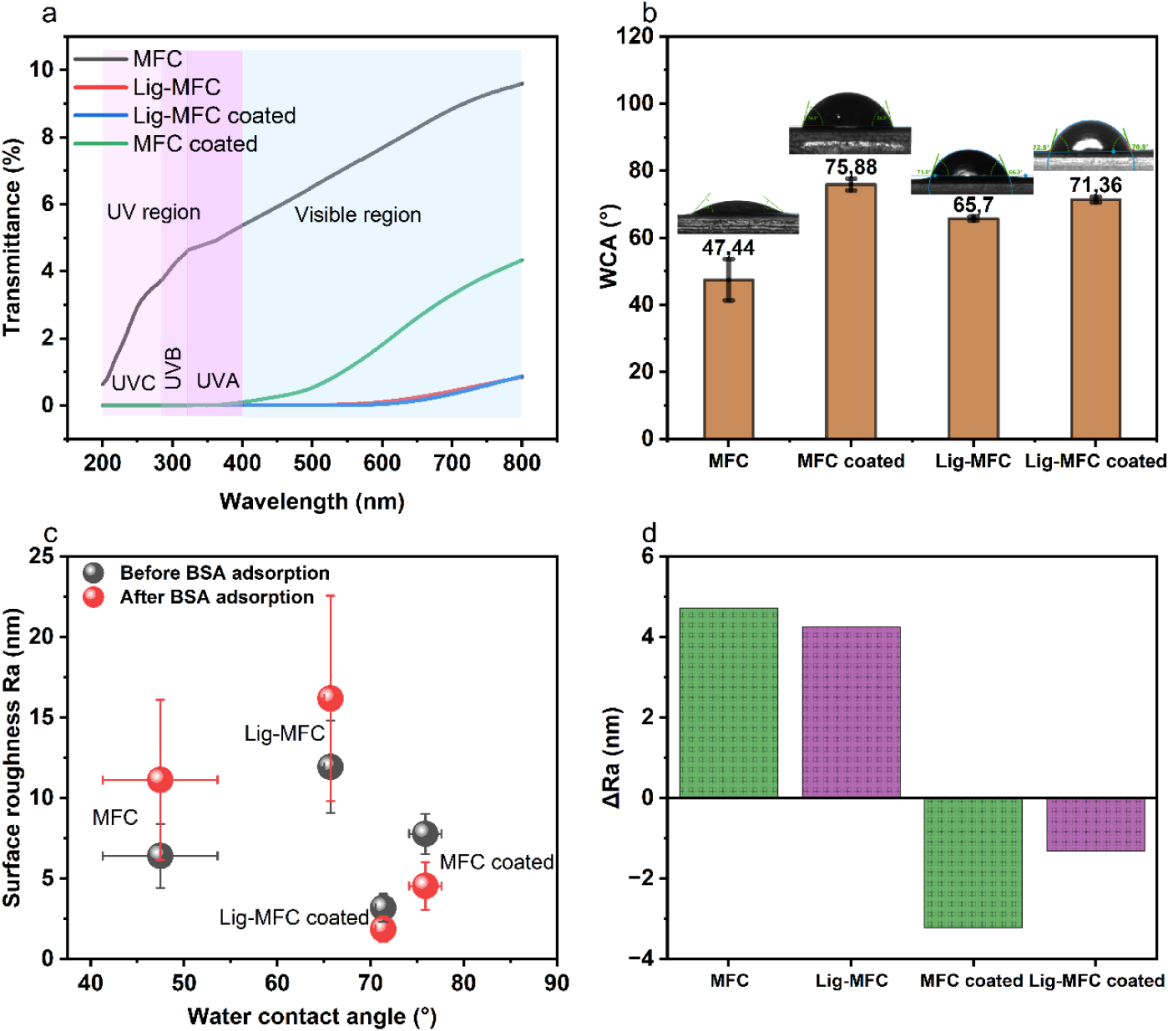
(a) UV–visible transmittance of MFC, Lig-MFC, and extractive-coated films. (b) Water contact angles of the four film types with representative droplet images. (c) Relationship between surface roughness and WCA, and (d) change in roughness (ΔRa) before and after BSA adsorption. These results show that the extractive coating reduces transmittance, increases hydrophobicity, and modifies surface topography.

Coating MFC and Lig-MFC with extractives further reduced the transmittance in both regions due to the presence of polyphenolic compounds, which absorb UV light and impart a brownish tint to the films. These compounds act synergistically with lignin, enhancing the UV-blocking efficiency. Dou et al. reported 99.999% UV light blocking for LCNF films produced from hot-water-extracted willow bark due to the combined effect of lignin and extractive chromophores. The extractives also increased the film density (see Table 1), contributing to higher light absorption and scattering. As expected, there is a trade-off between UV shielding and transparency; films with higher lignin or extractive content show stronger UV protection but lower visible-light transmission.^39^

**Table 1:** Grammage, Density, and Mechanical Properties of MFC, Lig-MFC, and Their Extractive-Coated Films

	Grammage (g/m^2^)	Density (kg/m^3^)	Tensile strength (MPa)	Young’s modulus (GPa)	Elongation at break (%)
MFC	36.0	930.2 ± 1.2	73.3 ± 1.9	4.2 ± 0.5	3.5 ± 0.6
MFC coated	49.9	1143.1 ± 0.5	119.2 ± 5.2	4.3 ± 0.8	11.0 ± 0.9
Lig-MFC	64.0	823.7 ± 2.6	45.2 ± 4.5	2.0 ± 0.3	4.5 ± 0.5
Lig-MFC coated	62.4	979.2 ± 2.1	53.1 ± 0.9	2.8 ± 0.3	7.5 ± 0.3

WCA is a key indicator of surface hydrophilicity or hydrophobicity, which directly affects the barrier performance, compatibility in composite applications, and wettability. The MFC film exhibited a WCA of 47.44° ± 6.16° (see Figure 5b), indicating a highly hydrophilic surface. This is characteristic of MFC, whose abundant hydroxyl groups promote hydrogen bonding with water molecules. Similar values (45–60°) have been reported for bleached CNF from kraft pulp or TEMPO nanocellulose films from bamboo.^9,40^ After coating the MFC film with the extractives, the WCA significantly increased to 75.88° ± 1.75° (Figure 5b), suggesting that the extractives contain hydrophobic moieties (confirmed by FTIR), which form a surface layer that reduces water affinity. The coating may also fill in surface pores, lowering the roughness and water adsorption capacity, as supported by the roughness data (Figure 5c–d). The Lig-MFC film showed a WCA of 65.7° ± 0.65°, higher than pure MFC, reflecting lignin’s lower polarity and more hydrophobic character. Its aromatic and aliphatic groups reduce the number of exposed hydroxyls.^40^ The Lig-MFC coated film reached 71.36° ± 0.83°, representing a moderate increase due to the different interaction between extractive, lignin, and the fibrillated network. The Ra value of MFC-coated films decreased after BSA adsorption, indicating that smoother surfaces resist protein binding and may reduce the rate of water uptake. Such a roughness reduction is known to enhance the apparent hydrophobicity on already low-energy surfaces, consistent with the Wenzel model. Interestingly, Figure 5c and Table S1 show that Lig-MFC films initially exhibited the highest surface roughness (Ra ≈ 11.94 nm), and after BSA adsorption, roughness increased further (ΔRa = ± 4 nm, Figure 5d). This suggests that their more open fibrillar structure facilitates BSA intercalation or adsorption onto micro- and nanoscale features, enhancing the surface heterogeneity. Across all samples, the increase in WCA corresponds to changes in both surface chemistry and roughness, particularly in the coated films, which show smoother and more hydrophobic surfaces (Figure 5c–d).

The combined improvements in optical, surface, and wetting properties can be explained by a synergistic mechanism arising from the interaction between the extractives and the fibrillar network. The extractives contain polysaccharide- and polyphenol-rich constituents that adsorb onto cellulose and lignin through hydrogen bonding, hydrophobic interactions, and π–π stacking. Their partial infiltration into the nanoscale voids between fibrils promotes microstructural densification and reduces the surface polarity, leading to smoother and less hydrophilic surfaces. This interfacial adsorption also enhances interfibrillar cohesion, contributing to the improved mechanical integrity observed in the coated films. In addition, the aromatic chromophores present in the extractives work together with lignin to absorb UV light more efficiently. Overall, the extractives act not only as an interphase modifier but also as a natural binder and functional component that tailors the optical, mechanical, and interfacial properties of the films.

### Mechanical Properties

The mechanical performance of the prepared films, including MFC and Lig-MFC films and their extractive-coated counterparts, is illustrated in Figure 6a–d and summarized in Table 1. As shown in the representative stress–strain curves (Figure 6a), the MFC-coated film demonstrated the most robust mechanical response, achieving the highest tensile strength (119.2 ± 5.2 MPa), followed by the uncoated MFC (73.3 ± 1.9 MPa), Lig-MFC-coated (53.1 ± 0.9 MPa), and uncoated Lig-MFC (45.2 ± 4.5 MPa) (Figure 6b). This strength enhancement is attributed to the densified structure imparted by extractive coating, coupled with the inherently finer microfibril network of MFCs, resulting in higher packing density (1143.1 kg/m^3^ vs 930.2 kg/m^3^ for coated and uncoated MFC, respectively), consistent with the positive effect of density on strength reported by Nair and Yan^41^

**Figure 6:**
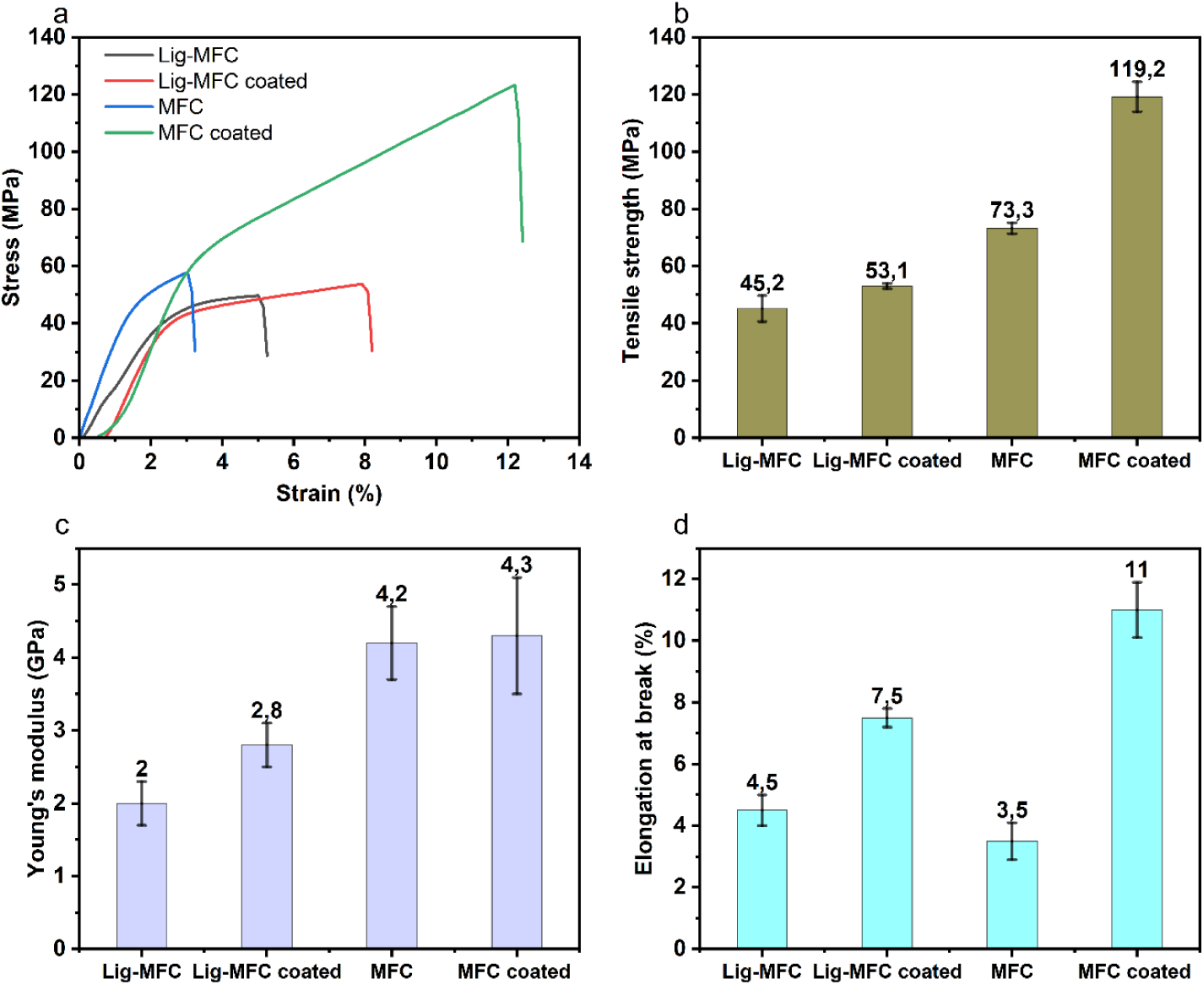
Mechanical properties of MFC, Lig-MFC, and their extractive-coated films: (a) representative stress–strain curves, (b) tensile strength, (c) Young’s modulus, and (d) elongation at break. Extractive coating increases strength, stiffness, and ductility, with error bars showing standard deviation (*n* = 3).

The Young’s modulus values (Figure 6c) mirror this trend: MFC-coated films exhibit the highest stiffness (4.3 ± 0.8 GPa), followed by MFC (4.2 ± 0.5 GPa), Lig-MFC-coated (2.8 ± 0.3 GPa), and Lig-MFC (2.0 ± 0.3 GPa). Interestingly, the extractive coating enhances the modulus even for lignin-rich films, likely by promoting better interfibrillar adhesion and reducing the porosity. The elongation at break (Figure 6d) improved significantly upon coating, especially for MFC-coated (11.00 ± 0.9%) compared to uncoated MFC (3.5 ± 0.6%), indicating a more ductile behavior induced by the flexible extractive layer, which may act as a natural binder—a mechanism also noted in bark-derived lignin-containing films.^42^

To benchmark these findings, Figure 7 provides a comparative assessment with reported cellulose- and lignin-rich films from the literature. In the tensile strength vs lignin content plot (Figure 7a), our MFC-coated film stands out with the highest strength at 0% lignin content, exceeding LC eucalyptus, and spruce bark CNF films.^43,44^ The Lig-MFC-coated sample (16.7% lignin) also surpasses similar lignin-rich systems like LCNF willow bark and LC bamboo, which often exhibit brittle behavior and limited tensile response due to high lignin levels (≥35%).^37,45^

**Figure 7:**
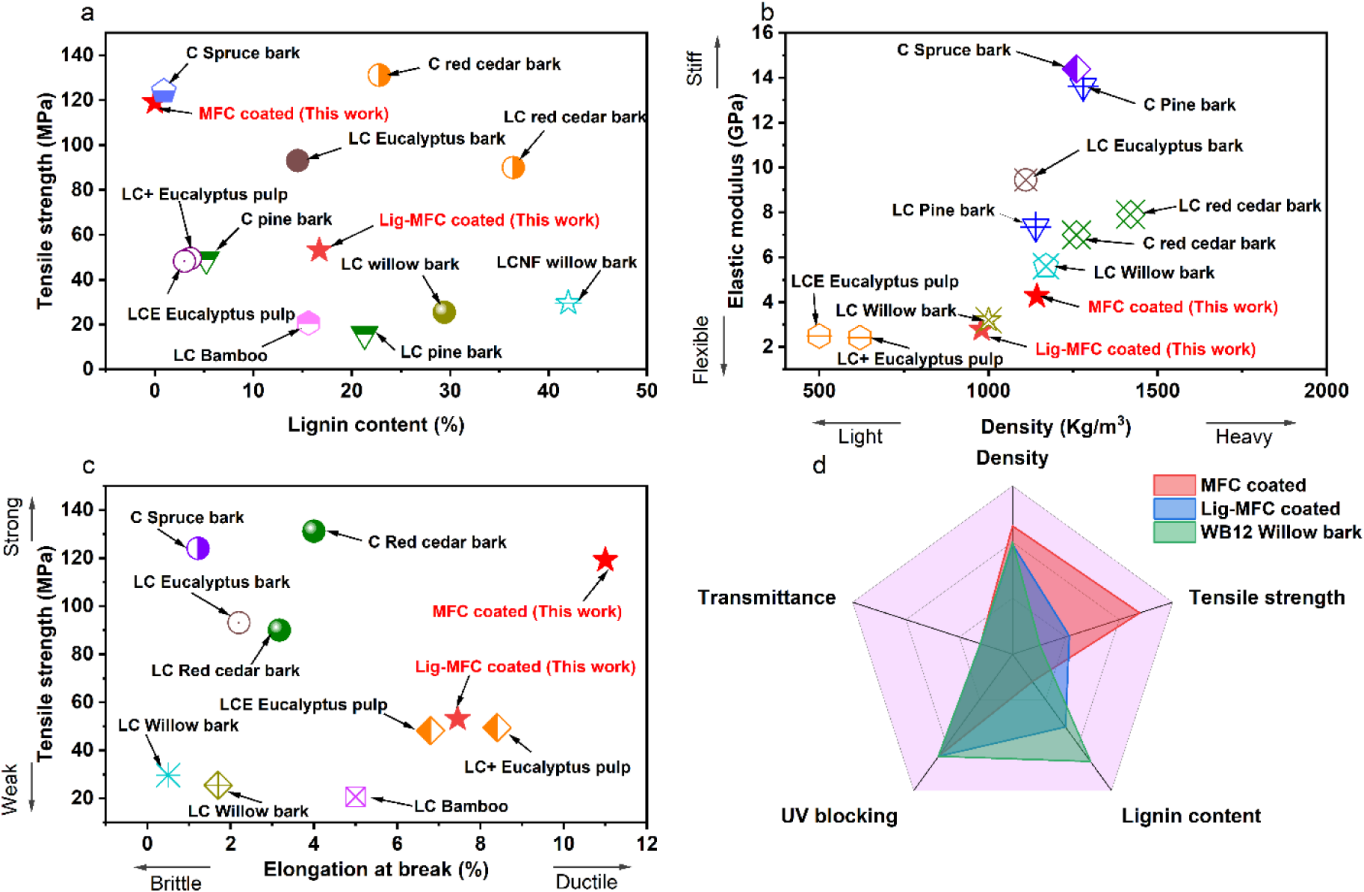
(a) Comparative plot of tensile strength versus lignin content for MFC and Lig-MFC-coated films against literature-reported lignocellulosic films from various sources, including red cedar bark (BNF, LNF), lodgepole pine bark (B15, UB15), eucalyptus (LMNFC, LCNF-cat, LCNF-enz), willow bark (LCNF, WB12), and spruce bark (SB-CNF). (b) Relationship between elastic modulus and density, (c) tensile strength as a function of the elongation at break, and (d) radar chart comparing other properties. In all the plots, C refers to either BNF, B15, and SB-CNF, LC refers to LNF, UB15, LMNFC, LCNF, LMNFC, and LCNF80–2, and LC+ corresponds to LCNF-cat and LCNF-enz.

In the modulus vs density plot (Figure 7b), our samples align along the expected trend: higher-density films demonstrate greater stiffness. This is consistent with results from bark-derived CNFs by Huang et al.^23^ and eucalyptus-based LCNFs by Supriyadi et al.,^44^ where densification of fibrillar networks played a central role in increasing modulus. The MFC-coated film’s high modulus at 1143 kg/m^3^, compared to Lig-MFC coated at 979.2 kg/m^3^, underscores the impact of compact packing in cellulose networks.

The tensile strength vs elongation plot (Figure 7c) confirms the unique position of the MFC-coated film, combining high tensile strength and ductility—characteristics rarely seen simultaneously in nanocellulosic films. This combination is often compromised in high-lignin materials, as seen in red cedar and willow bark CNFs.^23,37^ Finally, the radar chart (Figure 7d) summarizes the mechanical and structural balance of the tested films, highlighting the enhancement provided by extractive coatings not only in strength and modulus but also in flexibility.

Together, these results reinforce that lignin-containing nanocellulose films can achieve competitive mechanical properties if combined with appropriate extractive coatings and structural densification. The interplay of lignin content, density, and microstructure enables tailored strength–ductility trade-offs suitable for sustainable film applications.

### Environmental Life Cycle Assessment

To evaluate the environmental sustainability of producing the two qualities of microfibrillated cellulose (coated Lig-MFC and coated MFC), a comparative LCA study was performed. Benchmark incineration gave small climate savings (−0.12 kg CO_2_ equiv). Coated Lig-MFC gave −1.16, −1.87, and −6.99 kg of CO_2_ equiv, demonstrating substantial reductions when substituting kraft liner, PET, and LDPE, respectively. Coated MFC gave even higher savings: −1.45, −2.28, and −7.80 kg CO_2_ equiv when substituting kraft liner, PET, and LDPE (Figure 8). The mechanical strength of the coated MFC compensates for the additional chemicals used in the bleaching step and the losses of material during the bleaching process.

**Figure 8:**
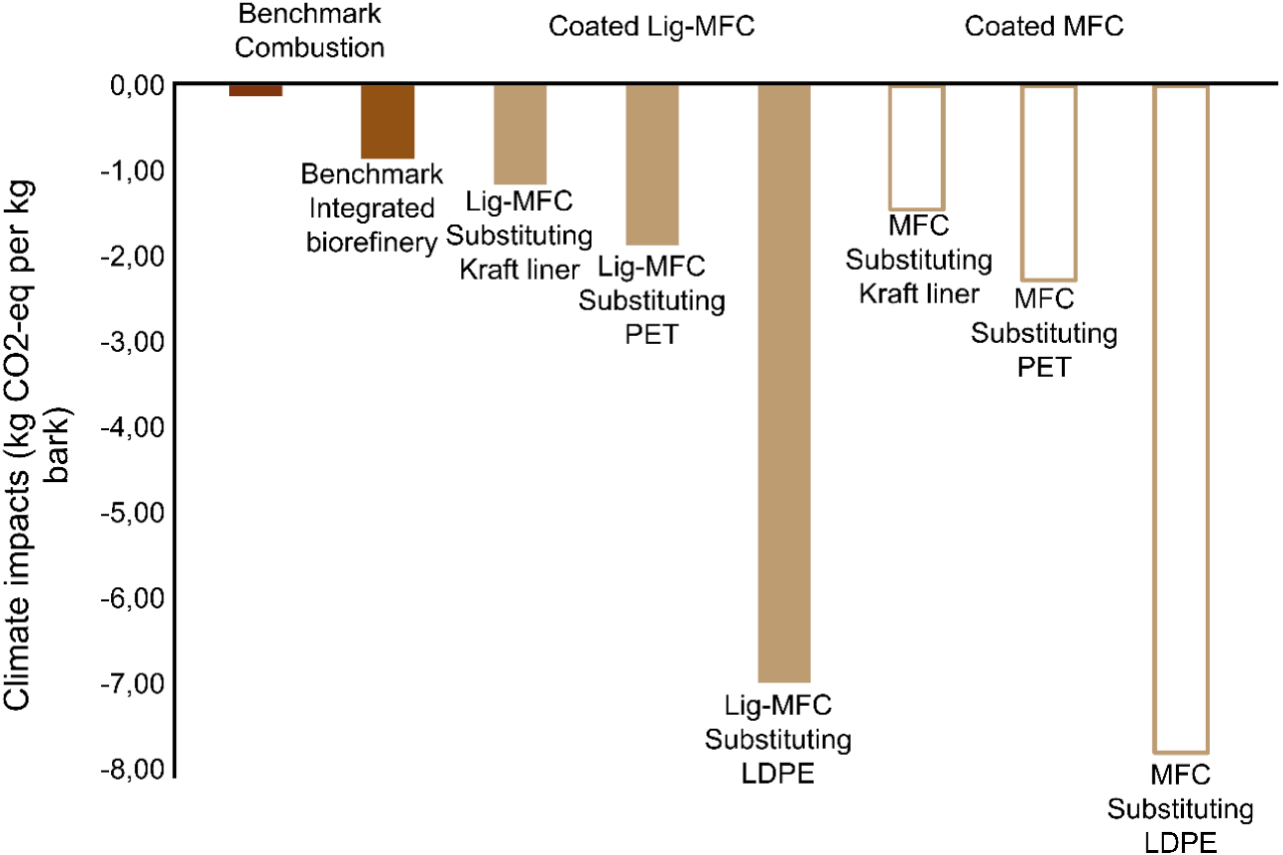
Climate impact assessment results comparing the incineration of bark with its valorization into multiple products, including coated Lig-MFC and coated MFC, for the substitution of conventional packaging materials within a pulp mill-integrated biorefinery facility.

Across the 17 other impact categories assessed, the integrated biorefinery scenarios consistently outperform bark combustion (see SI-2), showing lower toxicity, eutrophication, resource, and airpollution-related impacts. Beyond the comparison with combustion, it is also important to compare the three integrated substitution scenarios, in which coated Lig-MFC and coated MFC replace LDPE, PET, and kraft liner, with the integrated baseline representing the same biorefinery configuration. In these substitution cases, an additional process step for microfibrillated cellulose production increases energy and material demands, but this is outweighed by the benefits of displacing conventional LDPE, PET, and kraft liner, which are generally more impact-intensive across several impact categories. In the integrated baseline, pulp and hydrophilic extractives are not converted into MFC products but instead substitute market pulp and starch, leading to some avoided burdens. However, the integrated MFC substitution scenarios achieve larger overall environmental improvements as they exploit the same industrial infrastructure to generate higher-value products that replace more impactful fossil-based and kraft-liner materials.

These findings should be interpreted by considering several study limitations. The cradle-to-gate system boundary excludes the use-phase and end-of-life impacts of MFC-based packaging due to limited data availability. Furthermore, the function-based substitution factors rely on tensile strength proxies, which require empirical validation through prototype testing. Despite these limitations, this LCA study of both packaging material qualities (coated Lig-MFC and coated MFC) strengthens the previous assessments performed on the bark biorefinery concept by incorporating measured mechanical performance data, confirming the approach’s potential while highlighting areas for further optimization.

## Conclusion

In this study, we demonstrated an integrated valorization pathway for spruce bark by developing competitive lignocellulosic films from both the lignin-rich and extractive fractions of the biomass. The fractions were obtained using sequential fractionation in continuous flow, which is known to be advantageous for upscaling. We used a 100% bark-containing material that outperforms previously reported bark-based films in terms of overall mechanical integrity, hydrophobicity, and UV-shielding performance. This was achieved by combining the hydrophilic extractives as a natural coating with the fibrillated bark pulp, enabling a fully biobased film without external additives.

The extractive coating enhanced water repellency, improved UV-blocking capability, and contributed to a smoother and less hydrophilic surface while maintaining film flexibility. These improvements reflect the synergistic effect of the extractives and fibrillar network, as also supported by AFM and FTIR analyses.

The coated films also showed substantially strengthened mechanical behavior, indicating that the extractives promote interfibrillar adhesion and help densify the microfibrillar structure. When compared with literature benchmarks, the films developed here occupy a favorable position among bark-derived and lignin-containing materials, demonstrating their potential as viable, biobased alternatives for sustainable packaging.

Overall, the combination of improved mechanical performance, reduced hydrophilicity, UV-blocking capacity, and controlled surface structure demonstrates the potential of this approach for advanced biobased coatings and packaging materials. The comparative LCA study supports the previous LCA study of the biorefinery, suggesting substantial benefits in the valorization of bark instead of incineration. The methodology aligns with circular economy principles by valorizing all major spruce bark fractions, reducing biomass waste, and avoiding synthetic additives. This work illustrates that extractive-functionalized cellulose films offer a scalable, sustainable, and alternative to petroleum-based materials for packaging in applications where high strength, moderate moisture resistance, and UV shielding are required. This process offers, through its simplicity, the possibility of being integrated into a pulp mill biorefinery by using the existing technology and avoiding new process design.

## Supplementary Material





## Data Availability

All other supporting data are available from the authors upon request.

## Notes

SI-1: AFM image of Lig-MFC, an HPLC chromatogram for the hydrophilic extractive, GC–MS spectra of extracted lipophilics, modulus maps of MFC and Lig-MFC, peak force images of the films, surface roughness values, and consequential life cycle assessment of bark biorefining (PDF)
SI-2: Life cycle impact analysis (LCIA) results data (XLSX)
