# Supplementary material for: Valorization of Spruce Bark to Environmentally Sustainable Packaging Materials
Source: ACS Sustain Chem Eng. 2026 Jan 14;14(3):1596–607. doi: 10.1021/acssuschemeng.5c11166 (PMC12849044; doi:10.1021/acssuschemeng.5c11166)
Supplement: Supplementary file 1 [file sc5c11166_si_001.pdf]

Supporting information

Valorization of spruce bark to environmentally sustainable packaging materials

Houssine Khalili[1], Suthawan Muangmeesri[1], Lala Ramazanova[1], Léa Braud[2], Joseph S. M. Samec[1,3*], Aji P. Mathew[1,3*].

[1]Department of Chemistry, Stockholm University, Stockholm SE-106 91, Sweden.

[2]Department of Sustainable Development, Environmental Science and Engineering, KTH Royal Institute of Technology, Teknikringen 10B, Stockholm 100 44, Sweden.

[3]Stockholm University Centre for Circular and Sustainable systems, Stockholm University, SE-10691 Stockholm, Sweden.

*Corresponding authors: aji.mathew@su.se

* Corresponding authors: joseph.samec@su.se

Summary:

SI document contains **11 pages**

Figure S1 AFM image of Lig-MFC suspension; Figure **S2** HPLC chromatogram for the hydrophilic extractives**; Figure S3** GC-MS spectra of extracted lipophilics **; Figure S4** Nanomechanical modulus maps for MFC and for Lig-MFC. The peak force error images for the films before and after BSA protein adsorption.

Table S1 Surface roughness values (Ra) of the films before and after BSA protein adsorption; **S5** Consequential life cycle assessment of bark biorefining text including

Table S2 Substitution factors calculated from the average tensile strength of replaced materials; **Table S3**, Life cycle inventory scaled to the functional unit "1 kg of bark dry weight"; **Table S4** Overview of the LCA scores obtained for GWP 100. Values are expressed for each scenario in kg CO2-equivalent

Figure S1. AFM image of Lig-MFC suspension.

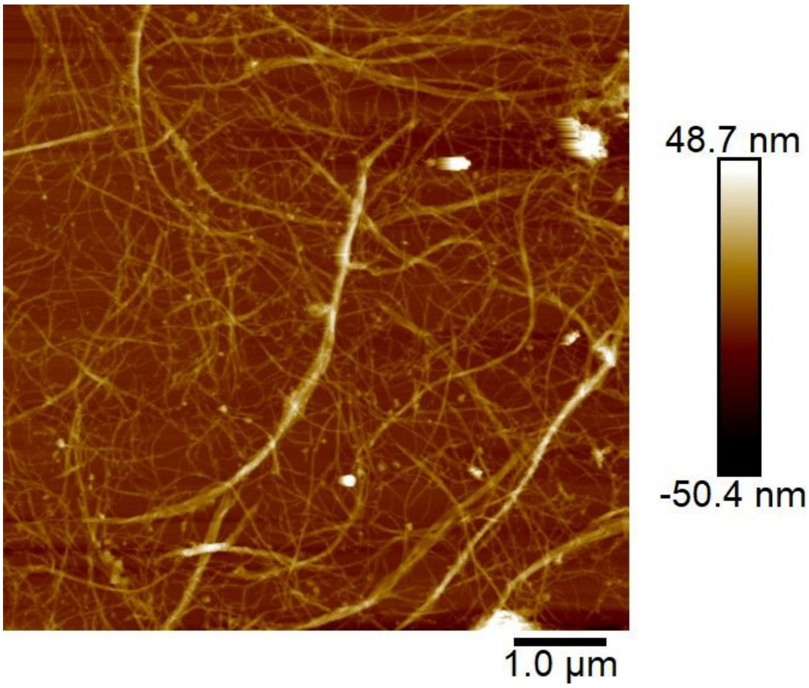

Figure S2. HPLC chromatogram for the hydrophilic extractives.

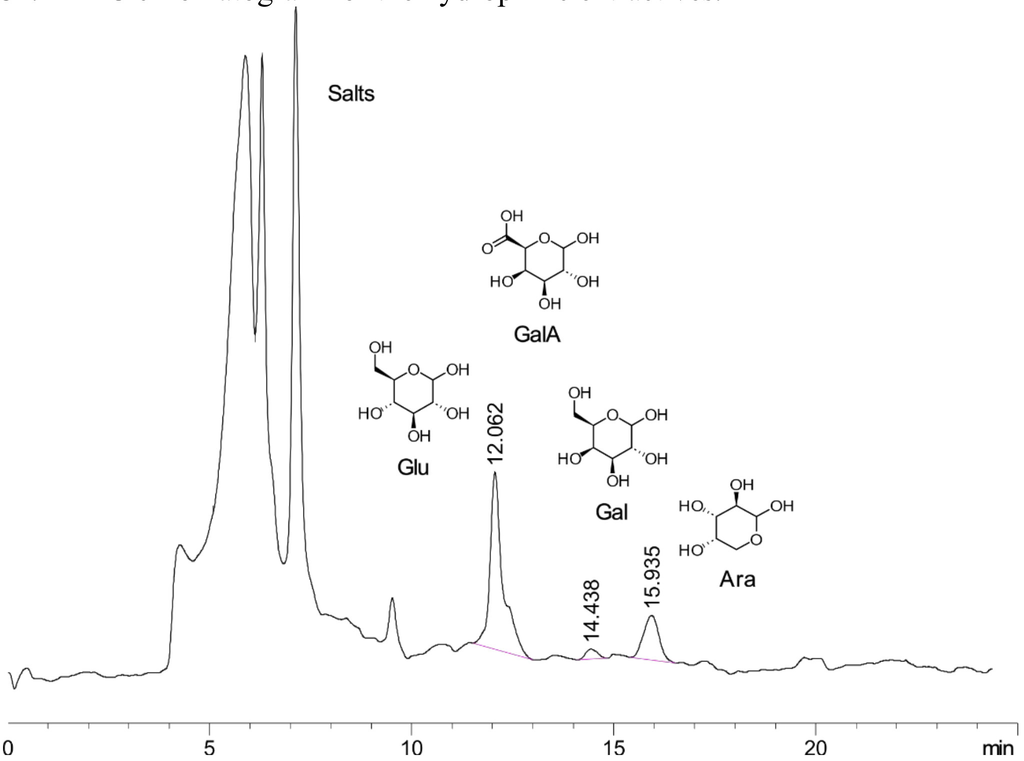

Figure S3. GC-MS spectra of extracted lipophilics

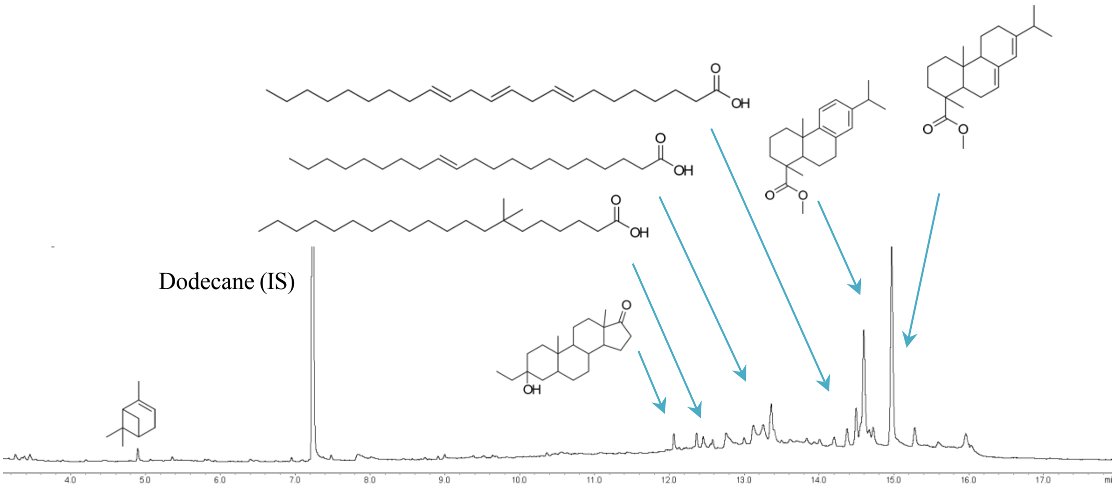

Figure S4. Nanomechanical modulus maps for MFC and for Lig-MFC. The peak force error images for the films before and after BSA protein adsorption.

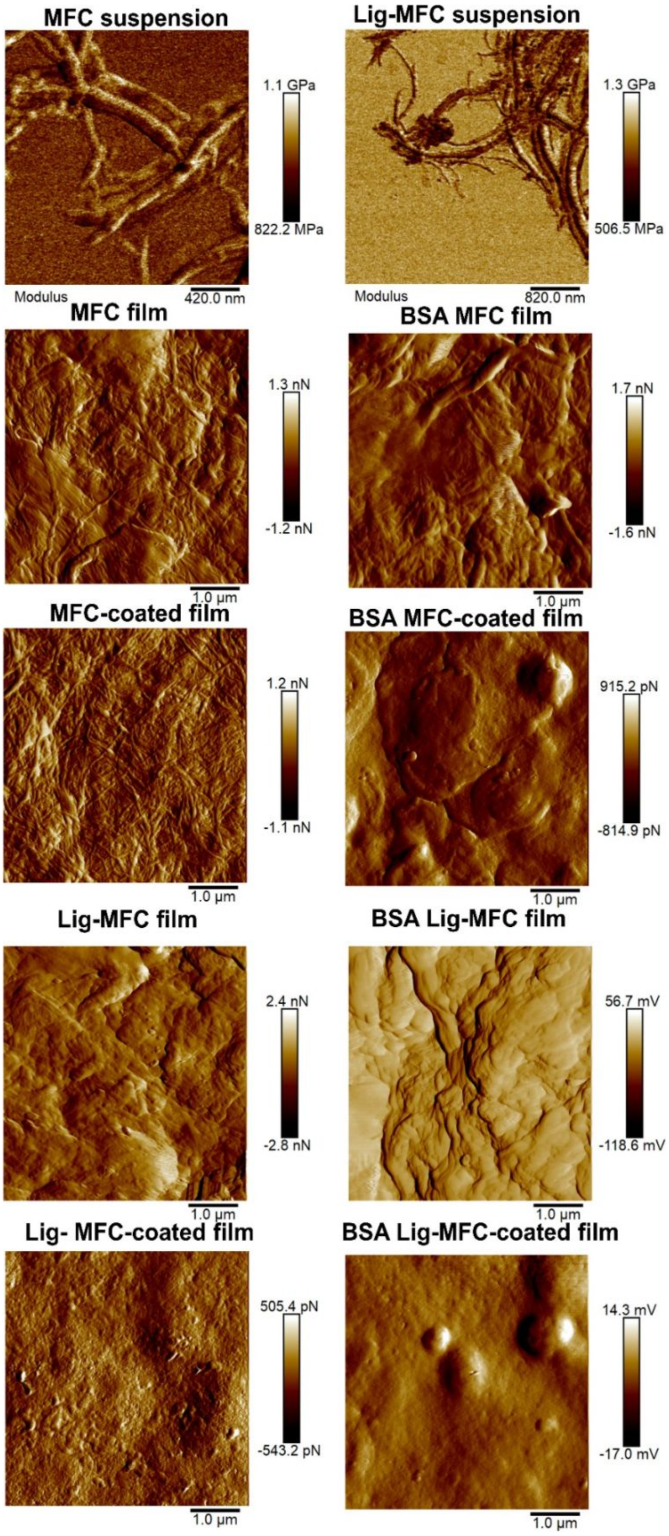
MFC suspension
1.1 GPa
822.2 MPa
Modulus
420.0 nm
Lig-MFC suspension
1.3 GPa
506.5 MPa
Modulus
820.0 nm
MFC film
1.3 nN
-1.2 nN
1.0 µm
BSA MFC film
1.7 nN
-1.6 nN
1.0 µm
MFC-coated film
1.2 nN
-1.1 nN
1.0 µm
BSA MFC-coated film
915.2 pN
-814.9 pN
1.0 µm
Lig-MFC film
2.4 nN
-2.8 nN
1.0 µm
BSA Lig-MFC film
56.7 mV
-118.6 mV
1.0 µm
Lig- MFC-coated film
505.4 pN
-543.2 pN
1.0 µm
BSA Lig-MFC-coated film
14.3 mV
-17.0 mV
1.0 µm

Table S1: Surface roughness values (Ra) of the films before and after BSA protein adsorption.

	**Surface roughness (Ra)**	
	**Before BSA adsorption**	**After BSA adsorption**
**MFC**	**6.41±1.98**	**11.13±4.97**
**MFC coated extractive**	**7.78±1.24**	**4.55±1.48**
**Lig-MFC**	**11.94±2.86**	**16.19±6.38**
**Lig-MFC coated extractive**	**3.18±0.87**	**1.86±0.79**

S5. Consequential life cycle assessment of bark biorefining

5.1. Goal and scope of the study

Goal: This LCA study compares the environmental impacts of biorefining bark against its conventional combustion (baseline scenario) and evaluates the consequences of valorizing a portion of the pulp and hydrophilic extractives into novel tensile materials, namely coated unbleached lig-MFC and coated bleached MFC, versus their direct substitution for pulp and starch on the markets. **Functional unit:** The functional unit (FU) chosen for this comparative study is "one kilogram dry weight of bark".

System boundaries: The system boundaries are limited to cradle-to-gate and exclude the environmental impacts from the use and end-of-life treatment of the biorefinery products.

Impact assessment: The life cycle impact assessment (LCIA) was conducted using the IPCC 2021 method, focusing on the Global Warming Potential (GWP) with a 100-year time horizon. In addition, the ReCiPe midpoint hierarchist (H) version 1.1 method was used to assess a broader range of environmental impact categories. The LCA calculations were performed using the Brightway LCA framework with ecoinvent v3.9. consequential. The parameterization of the foreground system was carried out with the lca_algebraic Python library. The Python scripts used to run the LCA are available in an open-source GitHub repository: https://github.com/leabrd/lcabark/tree/main/LCA2.

5.2. Scenario analysis

In this study, we compare the effects of producing coated unbleached Lig-MFC (**Lig-MF + coating)** versus coated bleached MFC (**bleached MFC + coating)** on the environmental impacts of the bark biorefinery. The production of **bleached MFC + coating** requires additional processing steps. However, the strength of the final product is higher meaning that it can also substitute "more" of conventional packaging material. The scenarios also compare biorefining versus combustion of bark, integration of the biorefinery processes, and different substitution strategies for the material produced from pulp and sugar extractives. The other parameters remain constant for all scenarios. The scenarios are listed below:

1) Bark combustion
2) Standalone biorefinery **Lig-MF + coating** substituting **kraft liner (x1.7)**
3) Standalone biorefinery **bleached MFC + coating** substituting **kraft liner (x4.0)**
4) Standalone biorefinery **Lig-MF + coating** substituting **PET (x0.8)**
5) Standalone biorefinery **bleached MFC + coating** substituting **PET (x1.9)**
6) Standalone biorefinery **Lig-MF + coating** substituting **LDPE (x4.2)**
7) Standalone biorefinery **bleached MFC + coating** substituting **LDPE (x9.5)**
8) Integrated biorefinery **Lig-MF + coating** substituting **kraft liner (x1.7)**
9) Integrated biorefinery **bleached MFC + coating** substituting **kraft liner (x4.0)**
10) Integrated biorefinery **Lig-MF + coating** substituting **PET (x0.8)**
11) Integrated biorefinery **bleached MFC + coating** substituting **PET (x1.9)**
12) Integrated biorefinery **Lig-MF + coating** substituting **LDPE (x4.2)**
13) Integrated biorefinery **bleached MFC + coating** substituting **LDPE (x9.5)**

The substitution values are based on the mechanical properties (tensile strength) of the **Lig-MF + coating** and **bleached MFC + coating**. The products substituted on the market are: kraft liner (20-40 MPa), PET (50-75 MPa), and LDPE (10-15 MPa).

1 weight of **Lig-MF + coating** corresponds to 53.1 MPa could substitute 1.7 weights of kraft liner, 0.8 weight of PET, and 4.2 weights of LDPE. The same calculations were made for **bleached MFC + coating** (see Table S2).

Table S2: Substitution factors calculated from the average tensile strength of replaced materials.

Product	Kraft liner<br><br>Tensile strength: 20-40 MPa (30.0 MPa)	PET<br><br>Tensile strength: 50-75 MPa (63.5 MPa)	LDPE<br><br>Tensile strength: 10-15 MPa (12.5 MPa)
Coated Lig-MFC<br>Tensile strength: approx. 53.1 MPa	1.7	0.8	4.2
Coated bleached MFC<br>Tensile strength: approx. 119.2 MPa	4.0	1.9	9.5

5.3. Life cycle inventory

Table S3 : Life cycle inventory data scaled to the functional unit "1 kg of bark dry weight", adapted from Braud et al. (2025)

	Amount	Unit
**Lipophilic extraction**		
Inputs		
Bark	1.00	kg
Ethyl acetate	0.002	kg
Electricity (grid mix or steam)	2.44	kWh
Outputs		
Lipophilic extracted bark	0.90	kg
Lipophilic extractives	0.02	kg
Energy (heat and power)	0.68	MJ
Ethyl acetate emission to air	0.002	kg
**Sugar extraction**		
Inputs		
Lipophilic extracted bark	0.90	kg
Water (from natural resources)	0.07	kg
Electricity (grid mix or steam)	5.00	kWh
Outputs		
Sugar extracted bark	0.68	kg
Hydrophilic extractives	0.22	kg
Energy (heat and power)	0.00	MJ
Wastewater	0.07	kg
**Pulping**		
Inputs		
Sugar extracted bark	0.68	kg
Water (from natural resources)	0.35	kg
Sodium hydroxide	0.00523	kg
Carbon dioxide (liquid or reciruclated)	0.124	kg

Calcium oxide	0.00629	kg
Heat (from biomass)	0.35	MJ
Electricity (grid mix or steam)	1.03	kWh
Outputs		
Pulp	0.41	kg
Phenol	0.17	kg
Energy (heat and power)	0.85	MJ
Limestone	0.00551	kg
Wastewater	0.35	kg
**Scenario A: Lig-MF + coating**		
Inputs		
Pulp	0.39	kg
Hydrophilic extractives	0.19	kg
Water	0.55	kg
Electricity (grid mix or steam)	2.175	kWh
Outputs		
Lig-MF + coating	0.58	kg
Water emissions to air	0.58	kg
**Scenario B: bleached MFC + coating**		
Inputs		
Pulp	0.41	kg
Hydrogen peroxide	0.024	kg
Ozone	0.066*0.059-0.0176	kg
Sodium hypochlorite	0.215*0.059-0.0293	kg
Hydrophilic extractives	0.076	kg
Water	0.28	kg
Electricity (grid mix or steam)	2.32	kWh
Outputs		
bleached MFC + coating	0.281	kg
Energy (heat and power)*	1.74	MJ
Water emissions to air	0.28	kg

**Energy produced from the combustion of 0.205 kg of residual biomass.*

5.4. Substitution values

Scenario A: Lig-MF + coating

- 0.99 kg kraft liner
- **OR** 0.46 kg PET
- **OR** 2.44 kg LDPE
- **AND** 0.02 kg pulp substitute replace 0.02 kg conventional pulp production
- **AND** 0.03 kg sugar extractives replace 0.03 kg starch

Scenario B: bleached MFC + coating

- 1.12 kg kraft liner
- **OR** 0.53 kg PET

- **OR** 2.67 kg LDPE

- **AND** 1.74 MJ heat (1 kg bark => 8.5 MJ, 0.205 kg => 1.74 MJ)

- **AND** 0.144 kg sugar extractives replace 0.144 kg starch (0.22 - 0.076 = 0.144 kg)

5.5. Overview of the ecoinvent datasets used

- market for electricity, high voltage SE (Sweden)
- market group for electricity, high voltage RER (Europe)
- ethyl acetate production RER (Europe)
- carbon dioxide production, liquid RER (Europe)
- market for sodium bicarbonate GLO (Global)
- market for sodium hydroxide, without water, in 50% solution state GLO (Global)
- quicklime production, milled, loose CH (Switzerland)
- treatment of wastewater, average, wastewater treatment Europe without Switzerland
- market group for municipal solid waste RER (Europe)
- heat production, wood chips from industry, at furnace 50kW CH (Switzerland)
- market group for tap water RER (Europe)
- market for hydrogen peroxide, without water, in 50% solution state RER (Europe)
- market for sodium hypochlorite, without water, in 15% solution state RER (Europe)
- market for ozone, liquid RER (Europe)
- Ethyl acetate (biosphere) (air)
- Water, river (biosphere) (natural resource, in water)
- Carbon dioxide, fossil (biosphere) (air)
- Carbon dioxide, non-fossil (biosphere) (air)

Datasets used for substitution:

- tall oil refinery operation GLO (Global)
- potato starch production DE (Germany)
- phenol production, from cumene RER (Europe)
- sulfate pulp production, from softwood, unbleached RER (Europe)
- market for limestone residue CH (Switzerland)
- market for kraft paper RER (Europe)
- polyethylene terephthalate production, granulate, bottle grade RER (Europe)
- packaging film production, low density polyethylene RER (Europe)

5.6. Overview of LCA results (see below)

Table S4 : Overview of the LCA scores obtained for GWP 100. Values are expressed for each scenario in kg CO_2-equivalent

Life cycle stage	S1	S2	S3	S4	S5	S6	S7	S8	S9	S10	S11	S12	S13
heat CHP bark	-0.12												
lipophilic extraction		0.45	0.45	0.45	0.45	0.45	0.45	0.01	0.01	0.01	0.01	0.01	0.01
sugar_extraction_withlosses		0.90	0.90	0.90	0.90	0.90	0.90	0.00	0.00	0.00	0.00	0.00	0.00
pulping_withlosses		0.35	0.35	0.35	0.35	0.35	0.35	0.03	0.03	0.03	0.03	0.03	0.03
coatedLigMFC		0.39	0.39	0.39				0.00	0.00	0.00			
heat (burd.)		-0.10	-0.10	-0.10	-0.10	-0.10	-0.10	-0.10	-0.10	-0.10	-0.10	-0.10	-0.10
heat (RB)		-0.02	-0.02	-0.02	-0.02	-0.02	-0.02	-0.02	-0.02	-0.02	-0.02	-0.02	-0.02
tall oil (sub.)		-0.04	-0.04	-0.04	-0.04	-0.04	-0.04	-0.04	-0.04	-0.04	-0.04	-0.04	-0.04
phenol (sub.)		-0.40	-0.40	-0.40	-0.40	-0.40	-0.40	-0.40	-0.40	-0.40	-0.40	-0.40	-0.40
pulp coatLigMF (sub.)		0.00	0.00	0.00				0.00	0.00	0.00			
starch coatLigMF (sub.)		-0.05	-0.05	-0.05				-0.05	-0.05	-0.05			
Kraft kiner coatLigMF (sub.)		-0.59						-0.59					
PET coatLigMF (sub.)			-1.30						-1.30				
LDPE coatLigMF (sub.)				-6.42						-6.42			
bleachcoatMFC					0.48	0.48	0.48				0.07	0.07	0.07
heat bleachcoatMFC (sub.)					0.02	0.02	0.02				0.02	0.02	0.02
starch bleachcoatMFC (sub.)					-0.24	-0.24	-0.24				-0.24	-0.24	-0.24
Kraft kiner bleachcoatMFC (sub.)					-1.50						-1.50		
PET bleachcoatMFC (sub.)						-1.50						-1.50	
LDPE bleachcoatMFC (sub.)							-7.03						-7.03
**Total**	-0.12	0.89	0.19	-4.93	0.74	-0.09	-5.61	-1.16	-1.87	-6.99	-1.34	-2.175	-7.69

Scenarios: (1) Bark combustion; (2) Standalone biorefinery Lig-MF + coating substituting kraft liner (x2); (3) Standalone biorefinery bleached MFC + coating substituting kraft liner (x6); (4) Standalone biorefinery Lig-MF + coating substituting PET (x1); (5) Standalone biorefinery bleached MFC + coating substituting PET (x3); (6) Standalone biorefinery Lig-MF + coating substituting LDPE (x4); (7) Standalone biorefinery bleached MFC + coating substituting LDPE (x11); (8) Integrated biorefinery Lig-MF + coating substituting kraft liner (x2); (9) Integrated biorefinery bleached MFC + coating substituting kraft liner (x6); (10) Integrated biorefinery Lig-MF + coating substituting PET (x1); (11) Integrated biorefinery bleached MFC + coating substituting PET (x3); (12) Integrated biorefinery Lig-MF + coating substituting LDPE (x4), (13) Integrated biorefinery bleached MFC + coating substituting LDPE (x10)
